# Disseminating Evidence-Based Psychological Treatments for Eating Disorders

**DOI:** 10.1007/s11920-015-0551-7

**Published:** 2015-02-07

**Authors:** Zafra Cooper, Suzanne Bailey-Straebler

**Affiliations:** Department of Psychiatry, Warneford Hospital, Oxford University, Oxford, OX3 7JX UK

**Keywords:** Eating disorders, Empirically supported treatment, Dissemination, Implementation, Scalable training

## Abstract

The research-practice gap is of concern in the treatment of eating disorders. Despite the existence of empirically supported treatments, few receive them. The barriers to wider dissemination and implementation of evidence-based treatment include clinician attitudes towards such treatments and the lack of sufficient numbers of suitably trained therapists to provide treatment. In this review we discuss these barriers in the context of the wider issue of the dissemination and implementation of psychological treatments and review the research with regard to the treatment of eating disorders. Particular emphasis is placed on examining recent efforts to expand the availability and reach of treatments by making treatment delivery and training more scalable. We highlight promising developments and areas where further research is needed.

## Introduction

The research-practice gap has been well documented in recent years. Treatment researchers and developers as well as those involved in formulating public policy have highlighted the problem in medicine generally and in the area of mental health in particular [1–5]. Despite the considerable progress that has been made in the development and empirical evaluation of psychological treatments, there is agreement that this gap needs to close if such treatments are to achieve their promise for public health benefit. The need to focus on both dissemination, defined here as the process of ensuring the adoption of treatments, and implementation, the process of translating these treatments into routine and persisting clinical practice, is further supported by evidence suggesting that even when patients do receive empirically supported treatments^1^, they are not always well delivered [3]. The research-practice gap is of particular concern in the treatment of eating disorders [6••].

## Eating Disorders—the Need for Dissemination and Implementation

The eating disorders, anorexia nervosa, bulimia nervosa, binge eating disorder, and their variants are serious disorders that are accompanied by significant impairment in physical and psychological functioning as well as in quality of life [7]. Over the past two decades, significant progress has been made in developing treatments for these disorders and evidence for their efficacy has been documented in both narrative and systematic reviews. In particular, cognitive behavioural therapy (CBT), a guided self-help form (GSH) of CBT, enhanced cognitive behavioural treatment (CBT-E) and interpersonal psychotherapy (IPT) are recommended for the treatment of bulimia nervosa, binge eating disorder and to a lesser extent the atypical eating disorders [8–13], with further support for CBT-E from a recently published study [14]. Treatment for adults with anorexia nervosa is less well supported [11–13, 15, 16] with a number of approaches including CBT-E and IPT showing some promise and newer treatments such as the Maudsley model for treatment of adults with anorexia nervosa (MANTRA) and cognitive remediation therapy (CRT) under further investigation [12, 13]. As yet, one specialist treatment has not emerged as clearly superior to the others, with a recently published study [17] being broadly consistent with this conclusion, although the version of CBT-E used differs from the published manual [18]. For adolescents with anorexia nervosa, family-based treatment (FBT) has received the most support [13, 19, 20] with preliminary support for CBT-E [13].

Yet despite the existence of these empirically supported treatments for eating disorders, they are not reaching those who need them. In part, this is due to individuals not seeking treatment or eating problems not being correctly detected in primary care [21, 22]. However, even when treatment is sought and offered, many do not receive empirically supported interventions [22–28]. In sum, the disconnect between research and practice is as much a problem in the eating disorders as it is for psychological treatments in general.

## The Nature of the Problem

The barriers to the dissemination and implementation of empirically supported treatments for eating disorders are not unique, with similar problems occurring in the case of most psychological treatments. Broadly, the barriers divide into those concerning attitudes and beliefs of practitioners and organisations about adopting and implementing new evidence-based treatments [5, 29, 30] and issues relating to the availability of and access to treatment [4, 31•]. Overcoming barriers of availability raises obstacles in the form of gaps in our knowledge about how treatments are best delivered and how training should be provided. We discuss these two main kinds of barrier in relation to treatments for eating disorders, concentrating particularly on recent efforts to expand the availability and reach of treatments by making treatment delivery and training more scalable. While the work on prevention of eating disorders clearly has an important role to play in the project of reducing the burden of disorder, it is not within the scope of the current review.

## Attitudes Towards the Use of Empirically Supported Treatments for Eating Disorders

While clinician and organisational attitudes that may be a barrier to the adoption of evidence-based psychological treatments in general have been fairly extensively discussed as noted above, the discussion of this issue in the field of eating disorder treatment is relatively recent. Studies have examined eating disorder clinicians’ attitudes and concerns about the use of empirically supported treatments and manual use in particular, and the attributes of those clinicians who are more likely to use such treatments.

### Concerns About the Use of Empirically Supported Treatments and Manuals

A number of recent studies of clinicians treating eating disorders have explicitly reported their reservations and concerns about the use of evidence-supported treatment. Two internationally based large surveys of eating disorder clinicians [23, 32] reported that concerns about the generalizability of research findings led clinicians to modify treatment in clinical practice, with only very small numbers reporting that they adhered closely to a manual. Consistent with these observations are reports that clinicians claim to use CBT but do not use it as their primary approach [33] or that they use an eclectic approach combining elements of evidence-based treatment with approaches that are not empirically supported [25]. Clinicians generally expressed the view that manual-based treatments were too rigid and constraining to be a good fit for their patients while lack of sufficient training and inconsistency with their own theoretical orientation were lesser concerns.

Two more recent studies concentrated on clinicians in two single countries. A survey of clinicians working in publicly funded specialist eating disorder clinics in the United Kingdom (UK) found that negative attitudes towards treatment manuals and the potential outcomes they might achieve were associated with three beliefs: that manuals did not stress the therapeutic alliance; that they did not contain clinical case examples, and that they were imposed by third-party payers [34]. An interview-based study of community clinicians treating eating disorders across one Canadian province found that CBT was not used because the majority of therapists did not regard it as consistent with their theoretical orientation or their personal clinical style. Many also reported that their clinical experience suggested that CBT was not effective, while a smaller proportion believed it was inflexible [26]. In contrast, the main reason therapists gave for not using IPT was lack of training.

Qualitative studies provide an opportunity to explore and understand clinicians’ attitudes in greater depth. One such study [27, 28] investigated the uptake of FBT amongst clinicians treating children and adolescents with anorexia nervosa and found that clinicians expressed concerns about implementing some specific aspects of the intervention (weighing, nutritional advice and family meals) and many reported the commonly held objection that “one size does not fit all”. They also cited organisational factors stressing that adoption of FBT would be facilitated by support from clinical managers. Another recent qualitative study [35] found that clinicians who had attended a training workshop on CBT-E were broadly positive about empirically supported treatment but did not think that it was appropriate to implement the treatment in its entirety in their routine practice.

### Characteristics Associated With the Use of Empirically Supported Treatments and Manuals

The literature on the characteristics, demography and psychology of those that use evidence-based treatment and manuals is fairly limited. Data from a large international survey of eating disorder clinicians and researchers suggests that manual use in the treatment of bulimia nervosa is more likely if clinicians are: younger; psychologists; involved in research and treating adult patients [36]. A study of clinicians who reported routinely offering CBT for eating disorders in the UK produced broadly consistent findings in that older, more experienced and more anxious clinicians reported using fewer of the core elements of evidence-supported CBT [24].

Two further studies report on the relationship between clinician’s emotional characteristics and their attitudes towards the use of evidence-supported manuals or particular elements of evidence-based treatment. Perhaps unsurprisingly, those clinicians with negative attitudes towards manuals had higher scores on depressed mood [34] and concern about implementing certain key features of evidence-supported treatment amongst those offering CBT was related to clinicians’ anxiety levels, with older more experienced clinicians generally being less concerned about their use [37].

## Implications of Clinicians’ Understanding of the Empirical Evidence

Even when clinicians are broadly supportive of an evidence-based approach, they may have a variety of different interpretations about the nature of the existing evidence and about how it should be used in treating their patients. A belief that findings from research studies may have limited applicability for their patients often leads them to rely exclusively on their own clinical judgement rather than research findings and to combine eclectically both empirically supported and unsupported treatments with little regard for the potential disadvantages of this approach. Furthermore, a belief in the importance of the therapeutic alliance above the content of the protocol and particular concern about certain interventions and the effects they may have on their patients may also contribute to widespread lack of adherence to evidence-supported practice.

Some of these concerns may be addressed by highlighting research studies that have had relatively few exclusion criteria for trial entry (e.g., [38]). More importantly, recent effectiveness studies in routine clinical settings have reported results largely consistent with research trial findings [39–42], albeit it with higher drop-out rates. Nevertheless, more information is needed from controlled trials on the use of treatments in routine clinical settings. Relatively little is known about the key mechanisms of action of treatments for eating disorders. Such knowledge would enable us to preserve effective treatment elements and eliminate redundant ones when adapting or simplifying treatments for more widespread routine use. Ongoing work on mediators of change [43] will be helpful in this regard.

A solution often offered for overcoming attitudinal barriers has been further training and ongoing supervision. There has been relatively little research on how to train eating disorder therapists (see further discussion below). We suggest that training should focus both on acquiring knowledge about treatments and their implementation and on a better understanding of the nature of the evidence supporting these treatments and how to interpret and use it.

## Expanding the Availability of Empirically Supported Treatment for Eating Disorders

In common with many other forms of psychological treatment, the evidence supporting treatments for eating disorders is derived from studies in which treatment is delivered individually or in small groups by highly trained mental health professionals meeting with patients in a face-to-face setting. Furthermore, research findings generally relate to a specific eating disorder diagnostic category. This is consistent with what is still the dominant model of treatment delivery. However, this model of delivery has been criticised because it is unlikely to be sufficiently scalable to meet the needs of all those who need treatment [44••]. At the same time, it has been noted that such treatments are inherently difficult to scale up [45••]. In the eating disorders, two major problems are highlighted: even in resource-rich countries, there are major geographic and demographic inequalities in the availability of treatments [46], and the problem is even more acute in resource-poor countries; and, more fundamentally, there are not sufficient numbers of therapists appropriately trained to deliver these treatments [31•] nor is training readily available. A number of interesting initiatives have been proposed to address these two problems, only some of which have been tried and tested within the field of eating disorders.

**Fig. 1:**
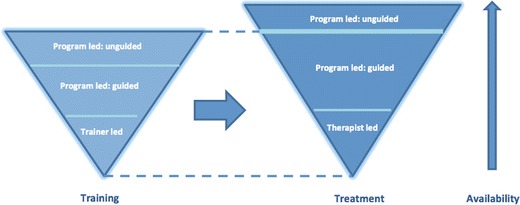
The relationship between scalable treatment and scalable training

A recent commentary detailing a “road map” for closing the research-practice gap suggests that initiatives to increase the availability of empirically supported treatments may be considered under two broad headings, those that remain essentially therapist led and those that are programme led [45••]. In the former case, the treatment content is delivered by a therapist although content, delivery medium or method of therapist training (which itself may be either trainer led or programme led, as discussed below) may be modified to make treatment more widely available. In the latter case, treatment content is conveyed directly to the individual by a variety of means (including books, CD-ROMs, downloadable and online resources) and may or may not be accompanied by the help of a guide. These alternatives can be viewed as existing along a continuum of increasing scalability (see Fig. 1).

## Therapist-Led Treatment for Eating Disorders

### Changing the Mode of Treatment Delivery for Difficult to Reach Populations

Simply changing the mode of delivery of therapist-led interventions has the potential to reach populations in areas where there are few therapists as well as patient groups who, for a variety of reasons, would not or could not attend face-to-face sessions. Two such approaches are of note. A study comparing 20-week manual-based face-to-face CBT for the treatment of bulimia nervosa (BN) with the same treatment delivered via telemedicine found the latter treatment to be cheaper with no differences in treatment outcome [46, 47]. An ongoing non-inferiority study is investigating 20-week group CBT, delivered either in face-to-face form or as a moderated chat group delivered over the internet, in terms of the efficacy, acceptability, attrition rate and cost effectiveness of the two treatments [48]. While these approaches clearly have advantages in making treatment more available, they still rely on trained eating disorder therapists who are in short supply.

### Training More Therapists

Of course, the most straightforward solution to a shortage of appropriately trained therapists is to train more of them. This has been the solution adopted by a number of large-scale dissemination and implementation projects such as the Improving Access to Psychological Treatments (IAPT) initiative across England [49] and the programmes adopted by the Veterans Health Administration [50]. There is no such similar project dedicated to dissemination and implementation of eating disorder treatments, and besides, projects such as these rely on the availability of large-scale resources to provide training and supervision.

In general, the topic of therapist training has been relatively neglected in the research literature and similarly the related issue of the measurement of the outcome of training has also been largely overlooked [51]. This applies also in the case of eating disorders. Generally, it is agreed that at least three components of training are necessary: attending a workshop led by an expert in the treatment; detailed study of a treatment manual and, usually thought most important, practising the treatment with ongoing expert supervision [52, 53]. In treatment studies, there may also be feedback on therapy quality from experts listening to recordings of sessions. While indirect evidence from patient outcome suggests that this is an effective form of training, this method is time consuming and depends on the availability of scarce and costly experts to provide workshops and supervision.

For sufficient numbers of eating disorder therapists to be trained, it is necessary to develop and evaluate models of training that are potentially more scalable. Possible ways of achieving this are to investigate training a wider range of therapists, to explore whether therapists may be trained in skills that are more widely generalizable and, perhaps most importantly, whether it is feasible for training to be programme led rather than trainer led (see Fig. 1). It is worth noting that the empirical evaluation of different methods of training (in a move towards evidence-supported training) raises the question of measuring the outcome of training [54]. A detailed discussion of the assessment of the outcome of training is beyond the scope of the present review.

#### Who Should Be Trained?

When considering who should be trained, two approaches hold promise for increasing the availability of trained therapists. The first, the train-the-trainer model makes use of an expert trainer to pass on skills to a range of less expert trainers who in turn can train larger numbers of therapists [53]. The second, more radical task shifting approach, aims to train a wider range of potential therapists to take on tasks usually performed only by those with specialist mental health qualifications [44••, 55]. By devolving specialist roles to less specialist or non-specialist therapists trained in particular interventions, the pool of potential trainees is increased, and almost certainly, the overall cost of training is decreased.

These approaches have barely been investigated in the field of eating disorder treatment. A proof of concept study investigated the outcome of guided self-help for those with binge eating problems in an open clinical trial where the training and supervision of the graduate student therapists was provided by a master’s level clinical psychology graduate who had been trained by an expert [56]. Although this train-the-trainer model appeared to be a feasible and acceptable strategy and results were consistent with those reported in the literature for self-help treatment, it should be noted that this study investigated a programme-led approach (guided self-help) rather than a therapist-led approach. A similar approach has not been investigated for a therapist-led treatment. Of concern is the question of whether train-the-trainer approaches can meet the scale of the problem [57].

To our knowledge, the task shifting approach has not been systematically investigated in training eating disorder therapists. The obvious objection to this approach is that it risks compromising the quality of care provided. Reports from the area of general medicine and early indications from the use of this strategy in mental health interventions in resource-poor environments suggest that it may not [58]. As such, it is a method worth investigating in training eating disorder therapists.

#### What Should Therapists Be Trained to Do?

As noted earlier, the dominant model of treatment delivery requires that therapists learn a number of different evidence supported treatments to provide the appropriate intervention to fit particular diagnostic categories. It has been argued that the need to train in a variety of single-disorder approaches may be unrealistic [59]. Transdiagnostic treatments (e.g., [60, 61]) provide the possibility of learning one empirically supported treatment, which may be applied flexibly to a range of psychopathology rather than to a particular diagnostic category. Similarly, modular treatments and principle- or component-based approaches may offer similar advantages [62, 63], although these have not been systematically investigated in eating disorders.

As noted earlier, CBT-E, a treatment designed to be transdiagnostic in scope, has received support in several RCTs [14, 38, (Fairburn, Bailey-Straebler, Basden, Doll, Jones, Murphy, O’Connor and Cooper, submitted)] although support for its use with patients who are significantly low in weight is still preliminary [64].

#### How Should Therapists Be Trained?

Programme-led rather than trainer-led methods of training have the potential to reach many more trainees. As noted, treatment manuals tend not to be used by large number of therapists. The availability of a range of modern methods of communication has created the possibility of programme-led approaches that might have several advantages over book-based treatment guides. One such approach potentially capable of training large numbers of therapists is web-centred training [51, 65, 66]. Details of treatment, guidance about how it should be implemented and illustrations of key procedures can be provided in interactive form online or in the form of downloadable material. Advantages of web-centred training include trainees being able to work through the training programme at their own pace, return to key material should they wish and potentially view many acted illustrations of clinical interventions. The training could possibly be undertaken with the help of guides who are not expert clinicians and who do not provide clinical supervision. Rather, their role would be to help trainees use the training programme effectively by motivating them to complete the training and implement treatment with their patients. Alternatively, training could be undertaken independently without a guide and it is possible that at least a proportion of trainees could benefit from this entirely scalable form of training.

Web-centred training has not yet been evaluated in the training of eating disorder therapists. We have recently completed a large pilot study in which one hundred therapists from a range of professional groups received guided web-based training in CBT-E using a newly developed training website. Preliminary results suggest that the method of training is feasible and acceptable to trainees, with very few trainees failing to complete training. Our group is currently comparing guided web-centred training with unguided independent training in an ongoing randomised controlled trial.

## Programme-Led Treatment for Eating Disorders

Even if training becomes much more available than at present, programme-led self-help approaches have the advantage of being able to reach a wider range of those who need treatment in a cost-effective way, including those who might not seek it through the usual clinical routes [45••, 67]. Additional benefits include the individual being able to pursue treatment at their own pace and a sense of empowerment [68]. Self-help interventions that involve an individual independently following a treatment programme, presented in either book or electronic form without any further support, have the most potential for wide or even global reach. Guided self-help involving varying degrees of input from a mental health professional or guide also has such potential, limited of course by the nature and amount of guidance provided, the qualifications of the guide and the amount of training and supervision received.

### Guided Self-Help

There is good evidence to support self-help versions of a number of empirically supported psychological treatments [69, 70]. Most recent work has focused on guided self-help rather than unguided or pure self-help, although available research findings do not conclusively favour guided self-help over unguided self-help [31•, 71]. A detailed recent review of the use of self-help to treat eating disorders found that there was good evidence for the use of guided self-help based on CBT principles for the treatment of binge eating disorder, that the evidence was less good but promising for the treatment of bulimia nervosa and that it was contraindicated in the treatment of anorexia nervosa [31•]. There is much less consistent evidence that it is as effective as therapist-led treatment. However, from the point of view of reducing the unmet need for effective treatments for eating disorders, a smaller effect size that can be widely and reliably achieved may be of vital importance [4].

One question that needs further consideration is the extent to which programme-led guided self-help approaches for the treatment of eating disorders are indeed scalable. While guided self-help is generally delivered by a wider set of providers than those that offer treatment, self-help guides tend to be health professionals (e.g., nurses) or trainee mental health professionals (graduate clinical psychology trainees, psychiatry residents, etc.) [31•]. With a few exceptions (see below), it has frequently been offered in face-to-face settings or by one-to-one telephone contacts in the context of university based research studies or research clinics [72]. Furthermore, the training and supervision received by guides varies greatly and is often not reported [73]. It is instructive to note that that the optimal training proposed for implementing guided self-help involves training and ongoing supervision or patient monitoring. It is judged optimal because it is associated with the best outcome. Of course, methods discussed earlier such as task shifting and web-based training may contribute to making guided self-help more scalable than it is at present (see Fig. 1).

### Unguided Self-Help

Self-help treatments may be more scalable if delivered primarily through unguided electronic or book-based approaches. At present, internet access is not universal and in certain areas and, for certain populations, self-help books may still be the most easily and widely available option. However, unguided treatment also raises a range of ethical and legal issues, which will need careful consideration [74].

Three recent systematic reviews have been published investigating self-help treatments [75, 76, 77•] delivered primarily by electronic means. While two of these reviews concluded that the internet was a promising method of treating eating disorders and even a good alternative to individual treatment [76], the third review using National Institute for Health and Care Excellence (NICE) methodology was much more cautious, concluding that the status of e-therapy was still uncertain for BN and binge eating disorder (BED) and unknown for anorexia nervosa (AN) [77•]. The use of mobile devices (mobile apps) either as an adjunct to treatment or as a means to deliver treatment has yet to be systematically explored [77•, 78•]. As in previous studies of self-help interventions, studies varied to the extent that they provided added support from a health professional or other guide.

Finally, two recent studies investigated unguided or pure self-help using a self-help book [72, 79] in a diverse group of patients with BED in primary care. In neither study was unguided self-help better than the control conditions.

Newer more scalable e-therapy versions of self-help are in the early stages of development and might benefit from using the full range of modern communication resources rather than simply transferring book-based material to an online format. Given the potential of various forms of unguided self-help for widespread use, further research attention is merited. Developing effective self-help interventions that can be used with little or no expert support and determining for whom they are likely to be helpful is an urgent priority. In particular, it is important to specify the amount and nature of any additional support participants are given when following these programmes including specifying details of the training and ongoing supervision provided for guides.

## Conclusion

The well-documented unmet need for eating disorder treatment together with growing evidence that patients do not receive empirically supported treatments has focused research attention on ensuring that such treatments are more widely available and used. Over recent years, relatively more attention has been directed towards developing and evaluating a range of programme-led self-help approaches rather than on investigating ways of increasing the availability of therapist-led approaches and training. We suggest that both are needed.

While self-help treatment holds the greatest promise for reaching those in need of treatment, it has not been shown to be suitable for the full range of eating disorders. New programme-led treatment interventions need to be subject to the same rigorous evaluation as demanded for other psychological treatments, as well as a systematic examination of their costs and public health impact. It is important to note that when evaluating scalable treatment, or indeed training interventions, non-inferiority designs [78•] are likely to be important. For programme-led treatment interventions, more use of “direct-to-user” recruitment [77•] is desirable if such interventions are to be shown to be truly scalable. Finally, determining for whom programme-led approaches are suitable is a priority.

Given that therapist-led treatment will continue to be required, there is also an urgent need for evidence-based training. In particular, new programme-based training methods need to be developed and rigorously evaluated. Training needs to address the problem of training more therapists by exploring further the use of train-the-trainer models, task shifting and transdiagnostic protocols where possible. There is also a need to further understand therapists’ concerns about using empirically supported treatments and address these in training. Programme-led approaches such as web-centred training hold promise for making training more widely available, but it has to be established that they do not compromise the quality of training therapists receive. The outcome of such training needs careful evaluation together with a more detailed study of therapist competence and its relation to patient outcome. Finally, further research on developing measures of therapist competence is required.

## Footnotes

1 It should be noted that the terms evidence-based, evidence supported and empirically supported are all used in the literature. They are often used interchangeably, and we have followed this practice.
